# ¿Dónde Viven Los Latinos? The Types of Neighborhoods Older Latinos Live in and Their Implications for Diabetes

**DOI:** 10.1093/geroni/igaa057.2244

**Published:** 2020-12-16

**Authors:** Catherine García, Jennifer Ailshire

**Affiliations:** University of Southern California, Los Angeles, California, United States

## Abstract

The spatial distribution of Latinos in U.S. neighborhoods is highly patterned due to a complex set of social, cultural, and economic forces, which leads to the differential distribution of and exposure of resources and opportunities across space. However, less is known about the types of neighborhoods older Latinos live in and how it impacts their health. Using census tracts from the year 2000, we employed a latent class analysis to explore how social and socioeconomic characteristics cluster together to create distinct neighborhood typologies. These typologies were then combined with data from the 2006-2016 Health and Retirement Study (n=3,047). We found that Latinos were more likely to live in predominantly Latino neighborhoods with low socioeconomic status (SES) (40%), multiracial neighborhoods with moderate SES (27%), and predominantly white neighborhoods with moderate SES (15%). Latinos living in predominantly Latino neighborhoods with low SES were more likely to have diabetes than other neighborhood typologies.

